# Arboreal Ants Use the “Velcro® Principle” to Capture Very Large Prey

**DOI:** 10.1371/journal.pone.0011331

**Published:** 2010-06-25

**Authors:** Alain Dejean, Céline Leroy, Bruno Corbara, Olivier Roux, Régis Céréghino, Jérôme Orivel, Raphaël Boulay

**Affiliations:** 1 Centre National de la Recherche Scientifique, Écologie des Forêts de Guyane (UMR-CNRS 8172), Campus Agronomique, Kourou, France; 2 Clermont Université, Université Blaise Pascal, BP 10448, Clermont-Ferrand, France; 3 CNRS, UMR 6023, Laboratoire Microorganismes: Génome et Environnement, Aubière, France; 4 CNRS, EcoLab (Laboratoire d'Ecologie Fonctionnelle), Toulouse, France; 5 Université de Toulouse, UPS, INPT, EcoLab, Toulouse, France; 6 Estación Biológica de Doñana, CSIC, Sevilla, Spain; 7 Departamento de Biología Animal, Facultad de Ciencias, Universidad de Granada, Granada, Spain; University of Arizona, United States of America

## Abstract

Plant-ants live in a mutualistic association with host plants known as “myrmecophytes” that provide them with a nesting place and sometimes with extra-floral nectar (EFN) and/or food bodies (FBs); the ants can also attend sap-sucking Hemiptera for their honeydew. In return, plant-ants, like most other arboreal ants, protect their host plants from defoliators. To satisfy their nitrogen requirements, however, some have optimized their ability to capture prey in the restricted environment represented by the crowns of trees by using elaborate hunting techniques. In this study, we investigated the predatory behavior of the ant *Azteca andreae* which is associated with the myrmecophyte *Cecropia obtusa*. We noted that up to 8350 ant workers per tree hide side-by-side beneath the leaf margins of their host plant with their mandibles open, waiting for insects to alight. The latter are immediately seized by their extremities, and then spread-eagled; nestmates are recruited to help stretch, carve up and transport prey. This group ambush hunting technique is particularly effective when the underside of the leaves is downy, as is the case for *C. obtusa*. In this case, the hook-shaped claws of the *A. andreae* workers and the velvet-like structure of the underside of the leaves combine to act like natural Velcro® that is reinforced by the group ambush strategy of the workers, allowing them to capture prey of up to 13,350 times the mean weight of a single worker.

## Introduction

The diversification of ants closely tracked the rise of angiosperms, the appearance of which created more complex habitats compared to the gymnosperms that had previously dominated the flora. This was accompanied by an increase in the abundance and diversity of potential prey in addition to the possibility of feeding on plant exudates [1], [2]. Most arboreal ant species do not depend on their host plants to provide them with nesting structures. Plant-ants, however, live in an obligatory association with ‘myrmecophytes’ that do provide them with a nesting place in pre-existing cavities (domatia) such as leaf pouches and hollow stems or thorns, and frequently food in the form of extra-floral nectar (EFN) and/or food bodies (FBs) [3]. In return, plant-ants protect their host plants from defoliators through their predatory and/or territorial behavior [3]–[5]. Also, most arboreal ants, including some plant-ants, attend sap-sucking Hemiptera for their honeydew, so that the loss of sap is frequently compensated by the protection the ants provide from defoliating insects [3], [6].

Except for myrmecophytic *Acacia*, *Piper* and *Macaranga* that produce protein-rich FBs and whose mutualistic plant-ants do not hunt, other plant-related products such as carbohydrate-rich EFN, FBs and Hemiptera honeydew are comparatively poor in protein and amino acids [3], [7]–[10]. So, many arboreal ants have developed innovative ways of meeting these needs. Some species economize nitrogen as their workers have a thin cuticle and non-proteinaceous venom [11]; others rely on micro-symbionts to recycle nitrogen [12]–[14], while still others consume a part of their attended Hemiptera that thus do not proliferate [6].

Other species must hunt to satisfy their need for protein; however, since the availability of prey in the tree foliage is unpredictable and most prey are insects able to escape by flying away, jumping or dropping [5], some arboreal ants have hunting techniques that appear to be adaptations to this restricted environment. Indeed, the workers of most territorially-dominant species - and some plant-ant species - ambush in a group; a worker that has successfully immobilized an insect emits a pheromone to recruit nearby nestmates to help it to spread-eagle the prey [4], [5]. Among plant-ants, *Azteca bequaerti* and *Tetraponera aethiops* workers, hidden in their host plant domatia, react to the vibrations transmitted by an alien insect landing on a leaf, making it unnecessary for them to forage for prey [4], [15], while *Allomerus decemarticulatus* workers build a gallery-shaped trap to ambush prey [16].

**Figure 1:**
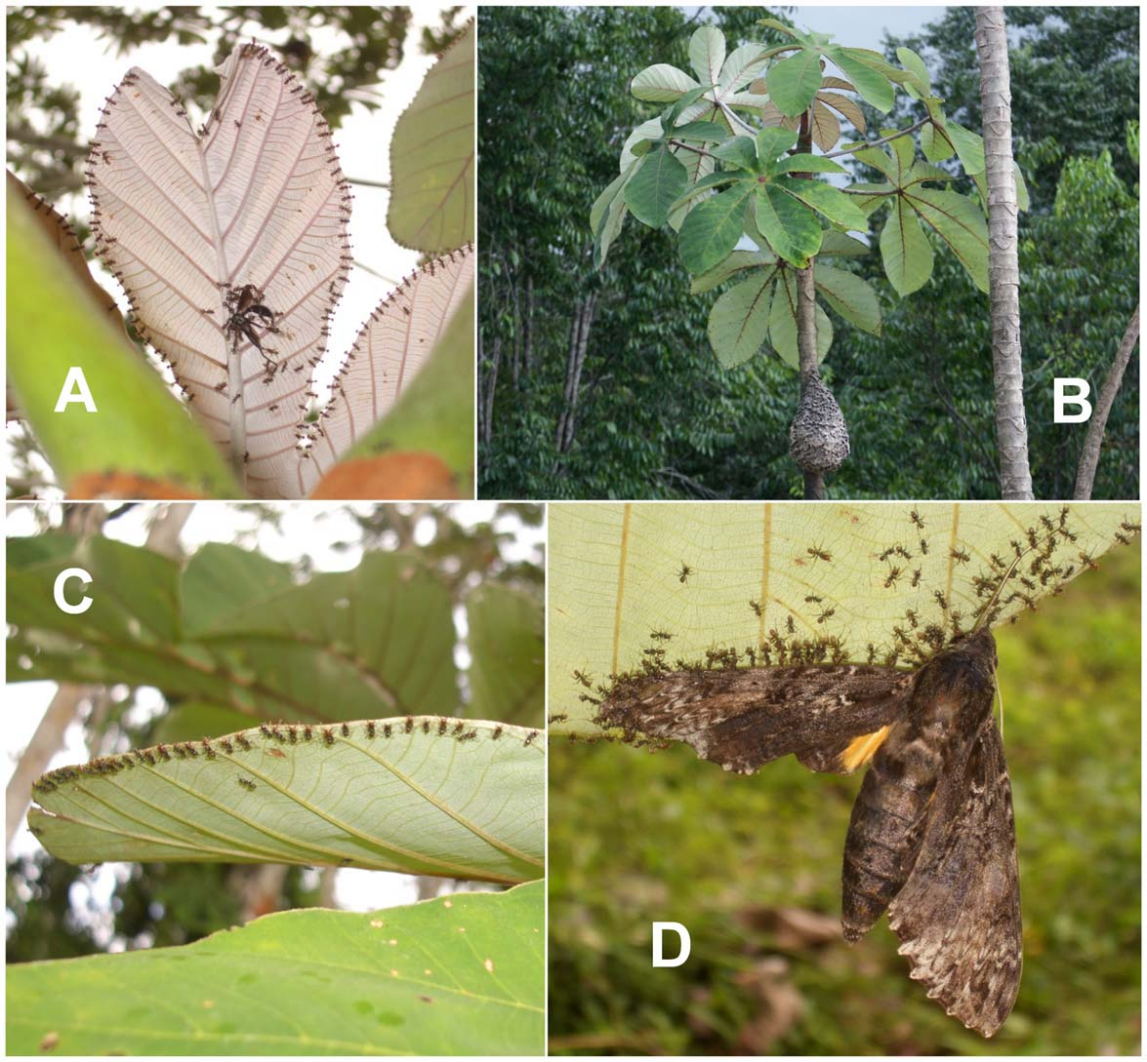
Carton *Azteca andreae* nest and group ambush technique. (A) A carton nest on a *Cecropia obtusa*. (B) Underside of a young *C. obtusa* leaf with numerous ambushing *A. andreae* workers placed side-by-side along the leaf margin. A black hymenoptera is spread-eagled near the principal vein. (C) A sphingid moth was captured during the night and was still struggling when we photographed it in the morning. (D) Detail of the position of ambushing workers.

An elaborate behavior was reported in *Azteca lanuginosa*, a generalist arboreal species of the Brasilian Cerrado whose group ambushing workers hide side-by-side under the leaves of shrubs with their mandibles wide open [17], [18]. Field observations suggested to us that *Azteca andreae* workers hunt in a very similar manner as *A. lanuginosa*, both on their host trees, the myrmecophytes *Cecropia obtusa* (Fig. 1A) and *C. palmata* (Cecropiaceae), and sometimes on the foliage of surrounding trees. These two *Cecropia* species house their guest ant colonies in hollow internodes, and provide them with FBs [10], [19]; however, after the incipient period, *A. andreae* workers build external, ovoid carton nests (Fig. 1B). Morphologically very similar, *A. lanuginosa* and *A. andreae* belong to the *aurita* group composed of species considered as temporary social parasites of other *Azteca* [19], [20]. Thus, when looking for a nest site after swarming, winged *A. andreae* queens likely select both the right *Cecropia* and *Azteca* species. Indeed, certain *Cecropia* species are not myrmecophytes, and only *A. alfari* and *A. ovaticeps* are associated with *C. obtusa* or *C. palmata* in the area studied.

Because we observed *A. andreae* workers capturing an 8-cm-long locust weighing 9.2 g - or ca. 7,100 times the weight (0.0014 g) of a hunting worker - on their host *C. obtusa*, we hypothesized that the *Cecropia* leaf structure could play a role in the capture of such a large prey. We therefore surveyed what kind and sizes of prey *A. andreae* workers can capture, studied the *C. obtusa* and *C. palmata* leaf structure, compared the workers' strength when holding onto different weights in five situations, and compared the successfulness of workers at capturing locust nymphs when hunting on *C. obtusa*, *C. palmata* and *Vismia latifolia* (Clusiaceae), with the latter serving as a control case.

## Results

### Prey capture by *Azteca andreae*

**Figure 2:**
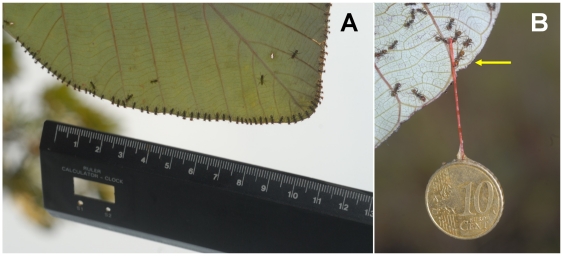
Illustration of the techniques used. (A) To evaluate the number of *Azteca andreae* workers per centimeter of leaf margin, we took pictures of the workers ambushing from beneath the *Cecropia obtusa* leaves while cautiously placing a ruler 1–2 cm away from the leaf margin so as not to perturb them. (B) To evaluate the strength of the workers, we used different weights glued to pieces of thread and placed the free end of the thread near an ambushing major worker. Here, three *Azteca andreae* workers are biting the end of a piece of thread glued to a 10-cent Euro coin; only one (arrow) is really holding onto the coin (4.11 g).

*Azteca andreae* workers occasionally hunt by patrolling their host tree foliage, but early in the morning – or, more frequently, at the end of the day and at night – they ambush prey by placing themselves side-by-side beneath the leaf margins with just their wide-open mandibles visible from above (Fig. 1C). After noting that they frequently occupy all of the leaf margins of their host trees, we evaluated the number of ambushing workers by multiplying the density of the workers by the total length of the leaf margins; for example, for the 10 leaves of a *C. obtusa* tree, we estimated that there were 4.4 workers per cm or ca. 8350 workers (see Fig. 2A).

We witnessed the capture of numerous insects, even large moths, that were attracted by the light of an ultraviolet (UV) lamp placed near the leaves of a tree whose guest *A. andreae* were ambushing in great number. The larger insects were successfully captured only when they were seized at the leaf margins (Fig. 1D; see also Video S1). This was confirmed by experimentally dropping 1.5-cm-long grasshoppers onto leaves ca. 2.5 cm from the margin. The vibrations triggered an alarm in the three to ten closest workers that collectively attacked the prey and drove it toward ambushing nestmates that then seized it and immediately flipped it under the leaves before spread-eagling it during 4 to 10 minutes. Meanwhile, new workers had replaced those involved in the prey capture by placing themselves side-by-side along the leaf margin. Once they had killed or stunned the prey, the ants collectively retrieved it by moving slowly toward the leaf petiole, and then toward the carton nest. Some grasshoppers were partially carved up on the spot.

By monitoring 12 *C. obtusa* during 22 non-consecutive days, we noted that the colonies captured on average 16.66±0.76 prey greater than 8 mm in length per day (N.B. smaller prey, not registered as they were too rapidly mastered and retrieved, were very numerous). The prey included a wide range of flying and jumping insects (see Table 1), the largest of which, a 10.5-cm-long *Tropidacris collaris* locust, weighed 18.61 g or 13,350 times the weight of a hunting worker.

**Table 1:** Different captured prey, their weight (or mean weight ± SE) and the ratio with the mean weight of a hunting worker (ca. 0.0014 g).

No. of cases	Prey	Weight in g	Ratio
30	Flies (Mucidae) (0.43±0.01 cm)	0.0165±0.0005	11.5
30	Winged termites (Isoptera, Rhinotermitidae) (0.4±0.01 cm)	0.0184±0.0001	13.2
30	*Cyclocephala s*p. (Coleoptera, Dynastinae) (0.9±0.02 cm)	0.099±0.003	71.07
3	*Otomerus* sp. (Lepidoptera, Saturnidae) (ca. 2 cm)	0.47±0.07	337.4
1	Unidentified locust species (Orthoptera, Acrididae) (2.4 cm)	0.77	552.7
2	*Rotchildia* sp. (Lepidoptera, Saturnidae) (ca. 4 cm)	1.14±0.035	818.4
1	Unidentified dragonfly (Odonata) (10 cm)	1.02	732.2
1	*Blatta* sp. (Dictyoptera, Blattodea) (ca. 4 cm)	1.42	1019.4
5	*Eacles* sp. (Lepidoptera, Saturnidae)	1.92±0.08	1378.3
1	*Xylophanes* sp. (Lepidoptera, Sphingidae) (5.7 cm)	1.95	1392.8
1	*Eumorpha* sp. (Lepidoptera, Sphingidae) (6.6 cm)	2.08	1485.7
1	Unidentified locust species (Orthoptera, Acrididae) (4.2 cm)	2.1	1507.5
1	Unidentified locust species (Orthoptera, Acrididae) (4.4 cm)	2.32	1665.5
1	*Isognathus* sp. (Lepidoptera, Sphingidae) (7.4 cm)	2.75	1964.3
1	Pseudophyllinae (Orthoptera, Tettigonidae) (4.8 cm)	6.36	4565.7
24	*Tropidacris collaris* (Orthoptera, Acrididae) (8.1±0.2 cm)	7.67±0.65	5506.1
1	*Tinacris albipes* (Orthoptera, Acrididae) (ca. 6.5 cm)	9.92	7121.3

### Workers' strength when holding onto a prey

**Figure 3:**
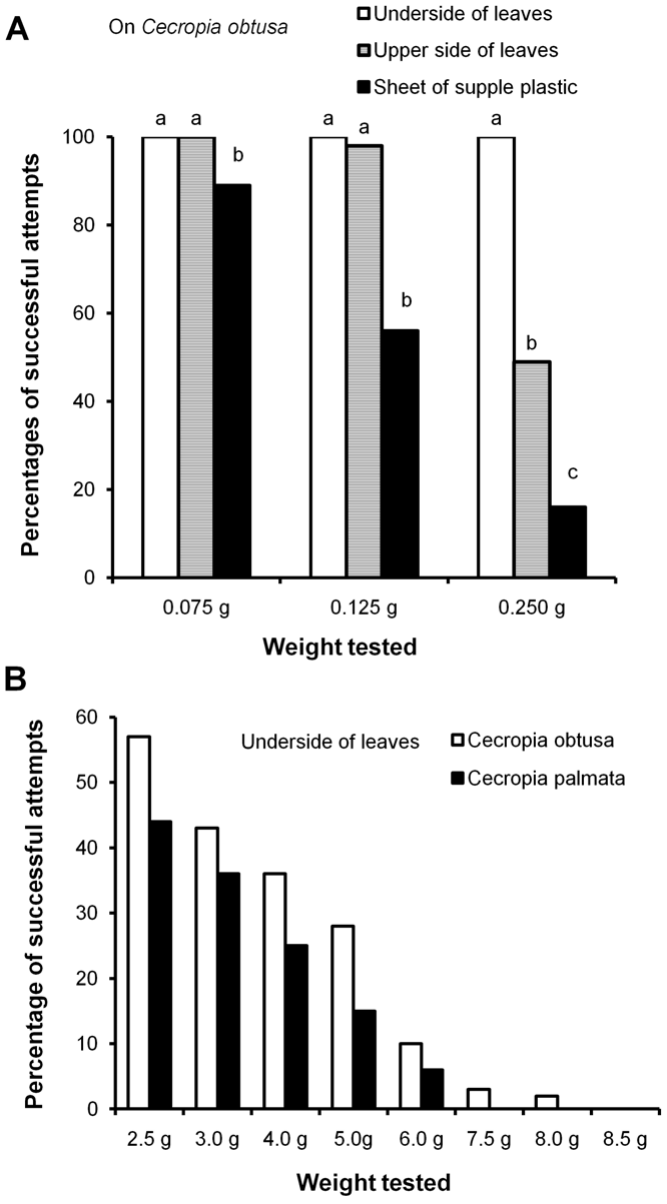
Percentages of cases when hunting *Azteca andreae* workers are able to hold onto different weights. (A) From the upper side and the underside of *C. obtusa* leaves, and from a sheet of supple plastic (Kruskal-Wallis test for 0.75 g: H_3,300_ = 12.4; P = 0.002; for 0.125 g and 0.250 g: H_3,300_ = 74; P<0.0001; Dunn's *post hoc* test for multiple comparisons: different letters indicate significant differences at P<0.01). (B) From the underside of *C. obtusa vs. C. palmata* leaves (Wilcoxon signed rank test: Z = 2.37; P<0.02).

Because the capture of such large and powerful prey was unexpected, we experimentally verified the workers' strength by placing the free ends of threads glued to different weights in front of individuals ambushing on a vertical part of a leaf. Tested individually, the workers immediately bit the end of the thread, and had enough grip to hold onto loads up to 8.0 g or 5,714 times their weight (Figs. 2B and 3).

**Figure 4:**
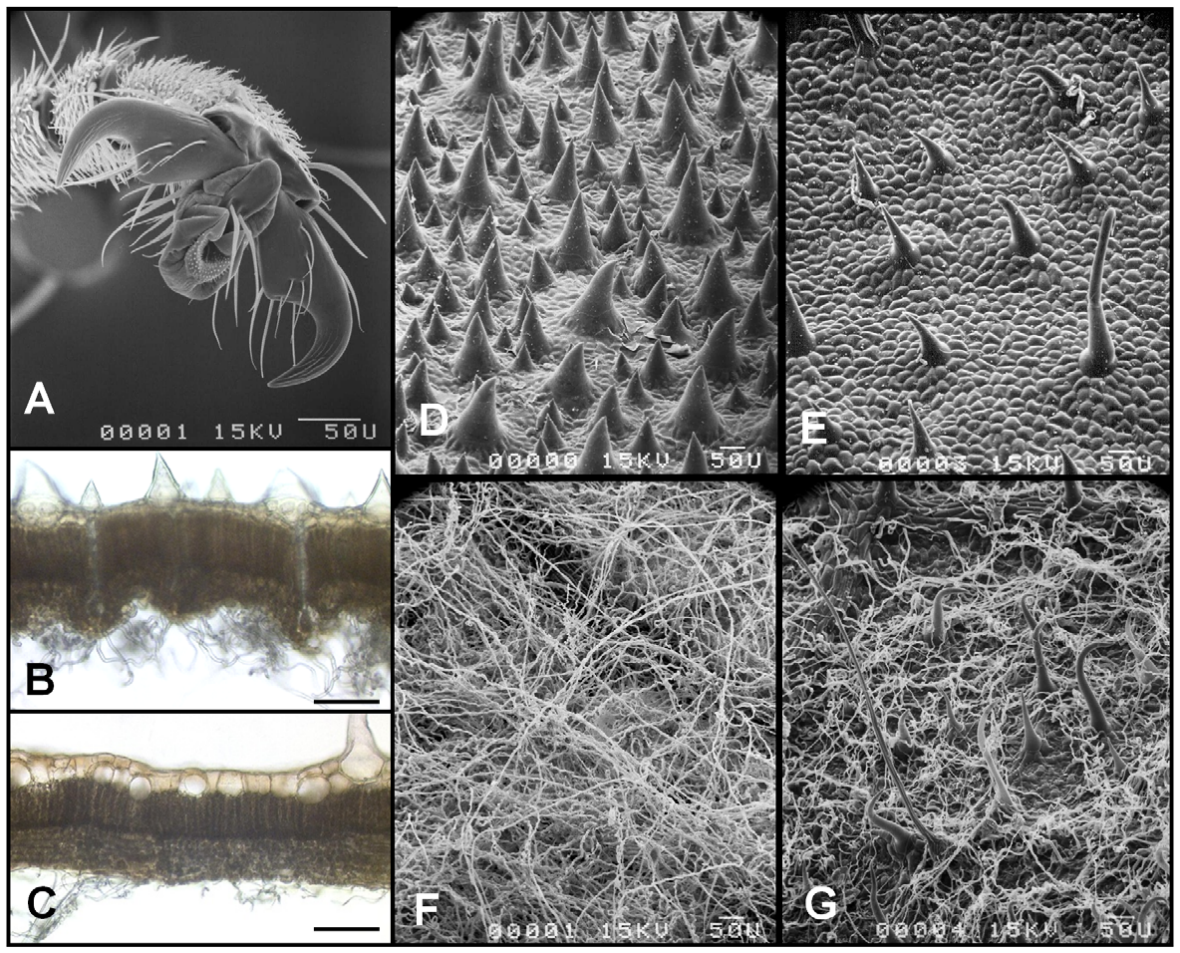
The hook-and-loop system permitting *Azteca andreae* workers to catch large prey. (A) Scanning electron micrograph of the hook-shaped claws of *A. andreae* workers. (B-C) Photomicrographs of unstained, 50 µm sections of *Cecropia obtusa* (B) and *C. palmata* (C); the upper side of the lamina is on the top. D-G- Scanning electron micrographs of the upper side (D–E) and underside (F–G) of the lamina of *C. obtusa* (D–F) and *C. palmata* (E–G). Long, thin trichomes characterize the underside of the leaves of both species, but with major differences in densities; whereas the upper surface of the leaves has short, wide trichomes – here, too, at different densities. Scale bars, 100 µm.

We noted a significantly higher number of successful cases when we tested workers situated on the very downy underside of *C. obtusa* leaves than when either on the rough upper side of these leaves or on experimental sheets of supple plastic (Figs. 3A and 4). The surface of the selected plastic does not allow ant claws to grip, so that the workers adhere thanks to their adhesive pads. Indeed, the velvet-like surface found on the underside of the *C. obtusa* leaves (Fig. 4) seems determinant in the workers being able to hold onto such weight. This is shown by the fact that ambushing workers from colonies associated with *C. obtusa* were significantly more effective than those from colonies associated with *C. palmata* (Fig. 3B), the underside of whose leaves is much less downy (Fig. 4).

### Capture of locust nymphs from four size classes and in three situations

**Figure 5:**
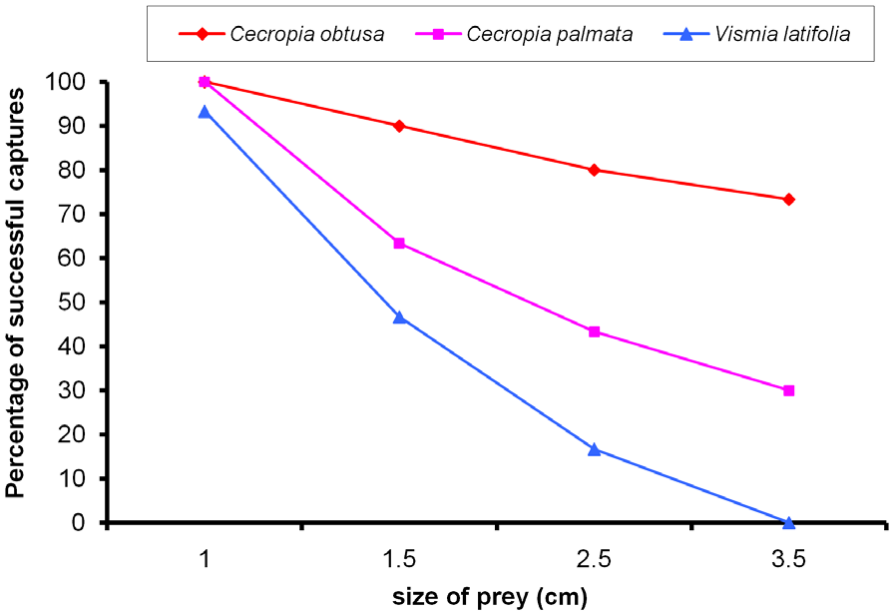
Capture of locust nymphs from four size classes on *Cecropia obtusa*, *C. palmata* and *Vismia latifolia*. The tests consisted in dropping 1 cm, 1.5 cm, 2.5 cm and 3.5-cm-long nymphs of the locust species *Tropidacris collaris* onto the upper surface of *C. obtusa*, *C. palmata* or *V. latifolia* leaves where groups of 12–15 *A. andreae* workers were hunting. These prey were dropped from ca. 5 cm in height at ca. 2.5 cm from the leaf margins.

We compared cases of the successful capture of locust nymphs from four size ranges when groups of 12–15 *A. andreae* were hunting on *C. obtusa*, *C. palmata* and *V. latifolia*. The latter tree species, the upper side of whose leaves is very smooth and the underside much less downy than those of the two compared *Cecropia*, served as a control case. We experimentally dropped the locust nymphs onto leaves ca. 2.5 cm from the leaf margins, and noted that both the tree species and the size of the locust nymphs had a significant effect on the ability of the ants to successfully catch the prey (p<0.001 in all cases; Fig. 5). Here, too, the leaf structure likely played a role as the effectiveness of the *A. andreae* workers, inversely related to prey size, decreased less rapidly when hunting on *C. obtusa* than on the two other tree species, and when hunting on *C. palmata* rather than on *V. latifolia*.

## Discussion

The effectiveness of the group ambush conducted by *A. andreae* workers is related to the structure of the leaves under which the workers hunt as the very downy underside of the blades facilitates both holding onto weight (Fig. 3) and capturing prey (Fig. 5). This permits a limited number of workers to hold onto large insects until their nestmates are able to help to spread-eagle these prey. This is particularly true for *C. obtusa* leaves (Fig. 4). In this case, the hook-shaped claws of *A. andreae* workers and the velvet-like structure of the underside of the leaves combine to act like natural Velcro® and is reinforced by the group ambush strategy of the workers. As a result, *A. andreae* workers can capture powerful prey up to 13,350 times their weight (i.e., equivalent to a 934.5-ton catch by a group of men each weighing 70 kg), while the host plant benefits from protection from even the largest defoliating insects.

“Velcro”, which has become a generic term derived from the French words ‘*velours*’ (for velvet) and ‘*crochet*’ (for hook), is a “hook-and-loop” fastener inspired by burdock seeds that are dispersed because they stick to mammal fur. Another case of a natural Velcro involved in an insect-plant interaction was recently described for the cone-shaped cells on the rough surface of flower petals that permit bumblebees to grasp the flowers while gathering nectar and pollen, and so to save energy by not having to beat their wings to stay on the flowers [21].

Published information on the maximum size and weight of the prey captured by arboreal ants is sparse. *Oecophylla longinoda* workers can capture large insects 20 to 50 times their weight, with this ratio exceptionally reaching 580 for a small bird captured and transported after it had fallen to the ground [22]. *Allomerus* workers use their gallery-shaped trap to capture insects up to 1800 times their weight [16].

As temporary social parasites, swarming *A. andreae* queens likely select nesting sites by looking for a host colony - here an *Azteca* species associated with myrmecophytic *Cecropia*. This behavior does not depend on the structure of the leaf blades of the host tree. Indeed, *A. andreae* colonizes both *C. obtusa* and *C. palmata*, and, if the *C. obtusa* leaf structure favors the capture of large prey, this is much less the case for *C. palmata* (Fig. 5). Also, their successful capture of the smaller prey (1-cm-long locust nymphs) was similar when tested on *V. latifolia* or on both *Cecropia* species (Fig. 5). Because prey of that size or smaller are the most frequently captured, they likely constitute the basis of the protein obtained by the colonies.

Due to the very similar group ambush technique used, the latter case is reminiscent of the one involving the generalist arboreal species *A. lanuginosa* whose workers forage on their host trees and on those situated in the vicinity [17], [18]. Indeed, their prey are 1.04 cm-long on average, although the capture of a ca. 4-cm-long lepidopteran was once observed [17]. Nevertheless, the range of sizes of prey captured by *A. andreae* is wider than that of *A. lanuginosa*. The capability of *A. andreae* workers to capture larger prey may be due to the number of workers involved in the ambush: 850 *A. andreae* workers per leaf on average *vs.* up to 90 for *A. lanuginosa* [17]. Leaf size was not a limiting factor for *A. lanuginosa* as groups of this ant do not occupy entire leaf edges [17]. Also, the leaf structure of the host trees plays a major role, so that prey capture by *A. andreae* is particularly facilitated when it is associated with *C. obtusa* (and much less so with *C. palmata*).

In conclusion, many ant species have adapted their predatory behavior to the constraints of their arboreal life. This study illustrates a three-fold context wherein a coordinated group hunting effort complements the workers' hook-shaped claws combined with the structure of the leaves of their host plant. Consequently, they use a very effective group ambushing technique permitting them to easily capture numerous insect prey, including large and powerful items, while protecting their host tree.

## Materials and Methods

### Study site and model system

This study and the preliminary surveys that permitted us to develop the appropriate experimental protocols were conducted between 2005 and 2009 along forest edges in zones situated around the field station at Petit Saut, Sinnamary, French Guiana (5° 03′ 39″ N; 53° 02′ 36″ W). *Azteca andreae* (*aurita* group [20]) constructs ovoid carton nests on the upper part of the trunk of the *Cecropia* tree (Fig. 1A), so that the nests are periodically rebuilt as the host tree grows. In French Guiana, *Cecropia obtusa* generally houses *A. alfari* or *A. ovaticeps* colonies in hollow stems, and provides them with glycogen-rich Müllerian bodies produced by the *trichilia* situated at the base of each leaf petiole and with lipid-rich pearl bodies produced on the underside of the leaves [10]. The same is true for *C. palmata* that develops on white sands (M.F. Prevost, pers. comm.).

### Predatory behavior

To evaluate the number of workers ambushing at one time, we took pictures of the workers ambushing from beneath the *Cecropia obtusa* leaves while cautiously placing a ruler 1–2 cm away from the leaf margin so as not to perturb the ants (Fig. 2A). To study prey capture, using four different colonies, we dropped prey (1.5-cm-long Tettigonid grasshoppers) onto the upper surface of the leaves from ca. 5 cm in height at ca. 2.5 cm from the margin (50 cases). Although they were intact and so able to jump, 49 out of the 50 tested grasshoppers were captured, and then retrieved.

Using a microscale (Mettler® AE 260), we individually weighed 300 hunting workers randomly gathered from three colonies (100 individuals from each colony), resulting in an average worker weight of 1.393±0.05 mg (±SE), so ca. 1.4 mg. They consisted of medium- to large-sized individuals.

We monitored 12 *C. obtusa* during 22 non-consecutive days and verified twice each day, at dusk and early in the morning, what prey were captured by the *A. andreae* workers. We thoroughly inspected the underside of the foliage, the trunk and the surface of the nests in order to note what prey were spread-eagled, and/or in the process of being slowly retrieved or cut up (generally on the nest). We gathered the most frequent and the largest prey for identification. The largest prey were weighed individually; whereas, for the most frequent prey, we gathered up to 30 individuals to obtain a mean weight (±SE). We then calculated the ratio between the weight of the captured prey and the mean weight of a hunting worker.

### Comparisons of the structure of the leaf epidermis

Pieces of the central lobe of the multi-lobed *C. obtusa* and *C. palmata* leaves were collected and immediately fixed in FAA (5% formalin, 5% acetic acid and 50% ethanol) before being stored in 70% ethanol. Cross-sections, 50 µm thick, were obtained using a vibrating microtome (Leica VT 1000S, Rueil-Malmaison, France). Unstained sections were observed using an inverted microscope (Leica DMIRBE, Rueil-Malmaison, France). Images were acquired with a CCD camera (Color Coolview, Photonic Science, Robertsbridge, UK). For scanning electron microscope (SEM) photography, pieces of leaves were dehydrated in 80, 90 and 100% ethanol and were critical point-dried with liquid carbon dioxide. The dried materials were attached with double-sided tape onto metal stubs, grounded with conductive silver paint and sputter-coated with gold/palladium. Observations were made using a scanning electron microscope (Hitachi C450) operated at 15 kV, and photographs were taken with Illford 125 ISO film.

### Testing the strength of the workers

To determine more precisely how much weight a single worker is able to hold onto, we glued one of the ends of pieces of thick thread onto different weights. The experiment consisted in taking a weight between the thumb and index finger and cautiously placing the free end of the thread near a major worker ambushing on a vertical part of a leaf (see Fig. 2B) and rather isolated from its nestmates so that it would not immediately recruit other workers. We considered the experiment to be valid when the workers could hold onto the tested weight for at least 5 seconds. If nestmates came to help the worker prior to the end of the 5-second period, the experiment was not taken into consideration.

We first conducted a series of experiments on two colonies sheltered by *C. obtusa* to compare the ability of major workers to hold onto small pieces of aluminum of varying weights (0.075 g; 0.125 g and 0.250 g) depending on whether the worker was situated (*i*) beneath the leaves (control), (*ii*) on the upper side of the leaves (no long trichomes, but a rough surface), or (*iii*) on a sheet of supple plastic (polypropylene) attached vertically to the tree trunk and selected because the texture of this surface does not permit ant claws to grip (smooth surface). In this case, the workers adhere thanks to their tarsal adhesive pads [23]. The same operation was repeated 100 times for each weight value and for each of the three situations. Comparisons were conducted using the Kruskal-Wallis test and Dunn's *post hoc* test for multiple comparisons. We also tested workers from two colonies sheltered by *C. obtusa* and two others sheltered by *C. palmata*, a species that develops on the white sands along the Guianese coast. Both *Cecropia* species shelter *Azteca alfari*, *A. ovaticeps* and, less frequently, *A. andreae*. The same operation was repeated 100 times for each weight value and each *Cecropia* species using major workers ambushing on the underside of the leaves. Comparisons were conducted using the Wilcoxon signed rank test (Statistica 7.1 software).

### Capture of locust nymphs from four size classes and in three situations

To compare cases of successful capture by *A. andreae* hunting workers according to different leaf structures, we conducted a study on *C. obtusa*, *C. palmata* and *V. latifolia*, with the latter serving as a control case. Indeed, among the plants on which we noted *A. andreae* workers in the process of hunting, the leaves of *V. latifolia* are relatively large (up to 20 cm in length and 8 cm in width), their upper side is very smooth and the underside much less downy than those of the two compared *Cecropia*.

The study was conducted on three *A. andreae* colonies for each compared tree species, and here, too, the tests consisted in dropping prey onto the upper surface of the leaves from ca. 5 cm in height at ca. 2.5 cm from the margin.

We used nymphs of the locust species *Tropidacris collaris* from four size ranges: 1 cm, 1.5 cm, 2.5 cm and 3.5 cm. These nymphs move in groups of up to 150 individuals of the same size and are therefore good candidates for such studies. The tests were conducted when groups of 12–15 *A. andreae* workers were hunting on *C. obtusa*, *C. palmata* or *V. latifolia*. For each size class of prey, we compared the number of workers involved in hunting using an ANOVA to be sure that during the tests on one plant species the number of workers involved in hunting was not greater than for the two other plant species. In all of the cases, the difference was not significant (P = 0.41; P = 0.88; P = 0.63 and P = 0.97 for tests conducted on locust nymphs of 1 cm, 1.5 cm, 2.5 cm and 3.5 cm, respectively; N = 30 cases in all situations).

Because our data were structured due to the fact that we used three individuals per tree species and each tree was used repeatedly (10 times), we used the Generalized Linear Mixed Model (GLMM) on R 2.8.1 (R Development Core Team, 2008) with the “glmer” function of the “lme4” package by Bates and Maechler. The GLMM was run on the rate of successful capture of prey with the binomial distribution option (binary results such as failure or success of capture), using the tree species and the size of the prey as fixed effects, and replicates as a random effect.

## Supporting Information

Video S1 Video showing the capture of a moth.
(8.87 MB MOV)
